# Emergence of novel porcine circovirus 2d strains in Thailand, 2019–2020

**DOI:** 10.3389/fvets.2023.1170499

**Published:** 2023-06-20

**Authors:** Chaitawat Sirisereewan, Thanh Che Nguyen, Taveesak Janetanakit, Roongtham Kedkovid, Roongroje Thanawongnuwech

**Affiliations:** ^1^Department of Veterinary Pathology, Faculty of Veterinary Science, Chulalongkorn University, Bangkok, Thailand; ^2^The International Graduate Program of Veterinary Science and Technology, Faculty of Veterinary Science, Chulalongkorn University, Bangkok, Thailand; ^3^Center of Excellence for Emerging and Re-emerging Infectious Diseases in Animals and One Health Research Cluster, Faculty of Veterinary Science, Chulalongkorn University, Bangkok, Thailand; ^4^Department of Veterinary Medicine, Faculty of Veterinary Science, Chulalongkorn University, Bangkok, Thailand; ^5^Center of Excellence in Swine Reproduction, Chulalongkorn University, Bangkok, Thailand

**Keywords:** porcine circovirus 2, PCV2d, mutation, recombination, pigs, Thailand

## Abstract

Porcine circovirus 2 (PCV2) has been recognized as a causative agent of porcine circovirus diseases (PCVDs) affecting the global swine industry. In this study, the genetic diversity of PCV2 strains circulating in Thailand between 2019 and 2020 was investigated using 742 swine clinical samples from 145 farms. The results showed PCV2-positive rates of 54.2% (402/742) and 81.4% (118/145) at the sample and farm levels, respectively. Genetic analysis of 51 Thai PCV2 genomic sequences showed that 84.3% (43/51) was PCV2d, 13.7% (7/51) was PCV2b and 1.9% (1/51) was PCV2b/2d recombinant virus. Surprisingly, the majority of the Thai PCV2d sequences from this study (69.77%, 30/43) formed a novel cluster on a phylogenetic tree and contained a unique ^133^HDAM^136^ on the ORF2 deduced amino acid sequence, which is in one of the previously identified immunoreactive domains strongly involved in virus neutralization. The PCV2b/2d recombinant virus also carried ^133^HDAM^136^. The emergence of the novel PCV2d strains predominating in Thailand was discussed. This study highlights the need for further investigations on the spreading of these PCV2d strains in other regions and the efficacy of current commercial vaccines.

## 1. Introduction

Porcine circovirus (PCV) 2, the causative agent of porcine circovirus diseases (PCVDs) affecting the global swine industry, is a non-enveloped single-stranded DNA virus containing a circular genome of 1766–1768 nucleotides (nt) (1) containing three main open reading frames (ORFs). Replicase protein encoded by ORF1 (*Rep* gene) is essential for viral replication (2). Capsid protein encoded by ORF2 (*Cap* gene) is a viral structural protein playing a significant role in the immunogenicity, virulence, and characteristics of the virus genotypes (3, 4). Finally, an ORF3 protein could induce apoptosis (4). Since its discovery, PCV2 has been recognized as a viral pathogen with a significant economic impact on the pig industry in various regions, particularly in North America, Europe, and Asia (5–7).

To date, PCV2 is classified into eight genotypes, PCV2a–h, based on the ORF2 nucleotide sequence (6). PCV2d is currently the predominant genotype worldwide (8), possibly due to selection pressure from the global PCV2 vaccination or the previously circulating PCV2 strains. In general, mutation at neutralizing epitopes might render the mutant virus less susceptible to the pre-existing antibodies (from vaccination or previously circulating viruses) (9). Hence, a further genetic shift from the current PCV2d strains was not unexpected. In this study, novel variants of PCV2d with a unique mutation at a previously recognized immunoreactive domain on the ORF2 were identified and found to rapidly dominate in Thailand. This finding may raise awareness for further investigations on the spreading of these viruses in other regions and the cross-protection with current commercial vaccines.

## 2. Materials and methods

### 2.1. Clinical samples

A set of 742 swine clinical samples, each collected from a different pig, were retrieved from the sample repository of Chulalongkorn University, Veterinary Diagnostic Laboratory (CU-VDL) and Diagnostic Laboratory of Large Animal Hospital and Students Training Center (DLSTC). The samples were originally submitted to CU-VDL and DLSTC as part of routine diagnosis from January 2019 to December 2020. These samples were obtained from 145 swine farms located in 18 provinces across different geographical regions of Thailand, primarily in the high pig-density areas in the Western, Central, and Eastern parts (Supplementary Figure 1). The corresponding data of these samples were also obtained, including sample types, sample collection dates, age groups or statuses of the pigs, clinical signs, and farm locations.

### 2.2. PCV2 detection and DNA sequencing

Viral DNA was extracted from the clinical samples by using the IndiMag Pathogen kit of viral RNA/DNA (Indical Bioscience, Germany) on the automated extraction platform. The obtained DNA was stored at −80°C until used.

For PCV2 detection, a real-time PCR assay was done using Luna^®^ Universal Probe qPCR master mix (NEB, MA, USA) with previously described protocol (10). The PCV2-PCR positive samples were further systematically selected for genetic characterization.

The sample selection process aimed to fulfill three criteria; (1) obtaining at least one PCV2 sequence from each of the six geographical regions of Thailand (the Northern, Northeastern, Central, Western, Eastern, and Southern regions), (2) including PCV2 sequences from both 2019 and 2020, and (3) acquiring a maximum of one PCV2 sequence from each individual pig. Whenever possible, samples with ct values lower than 30 during the PCV2 detection process were selected, to increase the likelihood of successful whole genome sequencing.

For genome sequencing, PCR amplicons were prepared and then submitted to the third-party sequencing company. The PCR assay was performed as previously described (11, 12). The PCR reactions were done using Onetaq^®^ 2x Master Mix (NEB, MA, USA). The PCR products were examined by 1% agarose gels and purified using NucleoSpin™ Gel and PCR Clean*-*up *(*MACHEREY-NAGEL, Germany*)*. The PCR products were then submitted to Celemics, Inc. (Seoul, Korea) for barcode-tagged sequencing. The obtained nucleotide sequences were assembled and validated with SeqMan and EditSeq software v.5.03 (DNASTAR Inc., Madison, Wisconsin, USA) and submitted to GenBank.

### 2.3. Sequence analysis

Classification of the Thai PCV2 sequences was done using a previously proposed phylogeny-based method (6). The Thai PCV2 sequences (*n* = 51) were aligned with a set of PCV2a-h reference sequences (*n* = 266, Supplementary Table 1) (6). In total, 317 sequences were used for phylogenetic analysis. Phylogenetic trees were constructed based on the Neighbor-Joining (NJ) algorithm using p-distance data. The classification was separately done using the complete sequences of genomic, ORF1, and ORF2 data. The tree was also reconstructed using the Maximum Likelihood method with a sequence of PCV1 (GenBank accession number: KJ408798) as an outgroup to confirm the topology.

For the initial PCV2d sequence analysis, a phylogenetic tree of complete ORF2 sequences of Thai PCV2d from 2010 to 2020 was built (*n* = 124). The data from 2010 to 2015 were retrieved from GenBank (*n* = 73, Supplementary Table 2) and the data from 2019 to 2020 (*n* = 51) were from this study. Nucleotide sequences were aligned and the phylogenetic tree was then constructed based on the NJ algorithm with the Maximum Composite Likelihood model (NJ-MCL method).

NCBI BLAST function (https://blast.ncbi.nlm.nih.gov) was performed using 19RBR58 ORF2 as a query sequence (6^th^ February 2023) to retrieve sequences with high similarity from GenBank database for further PCV2d ORF2 analysis. The dataset was named 19RBR58/BLAST (*n* = 440, Supplementary Table 3). The non-redundant version of 19RBR58/BLAST (19RBR58/BLAST/NR, *n* = 158) was used in phylogenetic analysis. A phylogenetic tree was constructed using NJ-MCL method.

Otherwise stated, all sequence alignment was done using the Clustal W algorithm (13) of BioEdit 7.2.5 (14). Phylogenetic tree construction was done using MEGA version 10.2.6 (15) with bootstrap analysis of 1,000 replications.

Recombination analysis was carried out using Recombination Detection Program (RDP, version 4.22) (16). Seven recombination detection methods were used; i.e., RDP, GENECOV, Bootscan, MaxChi, Chimera, SiScan, and 3Seq. A recombination event was accepted when it was detected by at least five methods with the *p* < 0.01. Bonferroni correction was applied. In the final step, the identified recombinant virus was re-analyzed with SIMPLOT software v. 3.5 by Bootscan methods (17) and a direct PCR sequencing covering the recombination breakpoint.

## 3. Results

### 3.1. PCV2d is the major genotype in Thailand

**Figure 1:**
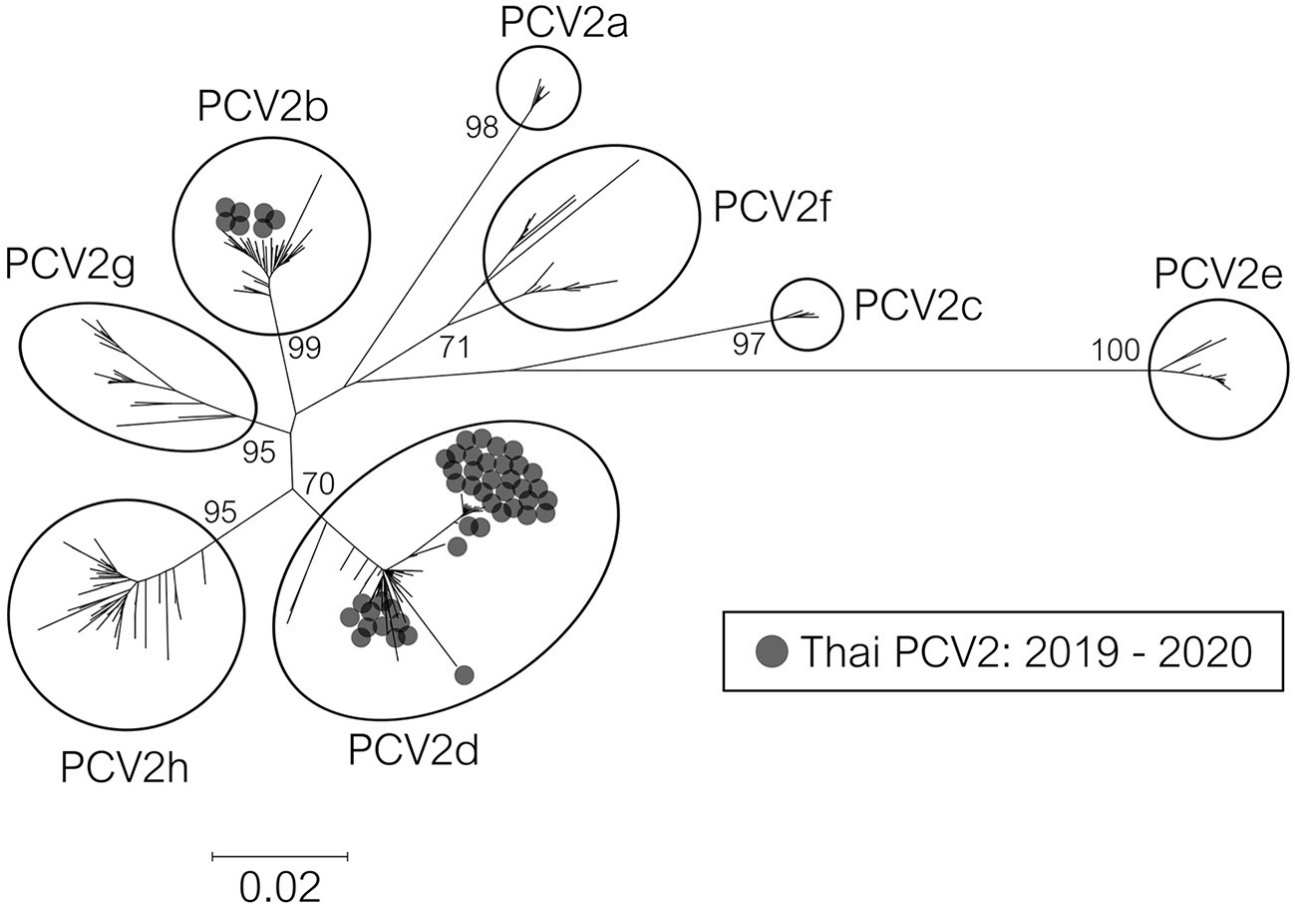
Phylogenetic tree of PCV2 ORF2 sequences from Thailand. The 317 complete ORF2 sequences were Thai PCV2 sequences (2019–2020) in this study and reference sequences from an available database (6). The tree was constructed using the Neighbor-Joining method with a p-distance model and bootstrapping at 1,000 replicates. Node labels indicate bootstrap values. The taxon position markers were adjusted to enhance readability.

PCV2 screening by real-time PCR was done on 742 pigs from 145 farms. The results are shown in Table 1. Overall, animal-level and farm-level positivity were 54.2% (402/742) and 81.4% (118/145), respectively. Fifty-one PCV2-positive samples (from 51 pigs) from 48 farms were genetically characterized. The nucleotide sequences were deposited in the NCBI GenBank database under accession no. OL677572–OL677622 (Supplementary Table 4). Phylogeny-based genotyping of the ORF2 data showed that the Thai strains were PCV2b and PCV2d (Figure 1), found at 13.73% (7/51) and 84.31% (43/51), respectively (similar results were observed when ORF1 or genome data were used). However, one strain, 19NPT29, was not grouped within any genotype clusters. At the farm level, PCV2b and PCV2d were found at 14.58% (7/48) and 87.50% (42/48), respectively.

**Table 1:** The prevalence of PCV2 in all tested samples during 2019–2020.

		**Types of samples**							**Prevalence at sample level**	**Prevalence at farm level**
**Periods**	**Group of pigs^†^**	**Pooled tissues**	**Serum**	**Feces**	**Semen**	**Oral fluids**	**Colostrum**	**Umbilical cords**	**Positive rate**	**Positive rate**
Jan-Dec 2019	Suckling	16/21	0/7	4/7	-	-	-	-	194/373 (52.0%)	62/81 (76.5%)
	Nursery	66/75	24/98	0/1	-	-	-	-		
	Growers	28/28	15/38	0/1	-	-	-	-		
	Breeders	2/4	14/48	1/1	1/10	-	-	-		
	Fetuses	23/34	-	-	-	-	-	-		
Jan-Dec 2020	Suckling	3/8	2/13	0/1	-	-	-	-	208/369 (56.4%)	66/74 (89.2%)
	Nursery	38/43	23/53	1/4	-	0/2	-	2/2		
	Growers	14/15	90/132	-	-	-	-	-		
	Breeders	0/2	10/38	-	1/12	-	2/2	-		
	Fetuses	22/42	-	-	-	-	-	-		
Jan 2019-Dec 2020		212/272 (77.9%)	178/427 (41.7%)	6/15 (40.0%)	2/22 (9.1%)	0/2 (0%)	2/2 (100%)	2/2 (100%)	402/742 (54.2%)	118/145 (81.4%)^‡^

^†^Suckling: < 4 weeks; Nursery: 5–8 weeks; Growers: 9–20 weeks; Breeders: boars, gilts, and sows.

^‡^The PCV2 positive farms were calculated from Jan 2019 to Dec 2020.

### 3.2. Novel PCV2d variants were identified and dominated among the PCV2d strains

Due to the high detection rate of PCV2d in this study, a phylogenetic tree was constructed to examine the genetic relationship between the current Thai PCV2d sequences (2019–2020) and the previously identified Thai PCV2d sequences (2010–2015). A cluster of PCV2d strains exclusively from 2019 to 2020 with a high bootstrap support was identified (data not shown). This cluster was named 19RBR58-like cluster, which accounted for 69.77% (30/43) of the PCV2d strains or 58.82% (30/51) of the PCV2 in this study. Percent nucleotide sequence identity of the 19RBR58-like cluster were as follows; genomic: 99.60–100, ORF2: 99.29–100, and ORF1: 99.58–100. Amino acid sequence identity was as follows; capsid: 99.15–100, and replicase: 99.36–100.

ORF2 nucleotide and amino acid sequence alignment were examined to identify a distinctive genetic characteristic of the 19RBR58-like cluster. A unique ^133^HDAM^136^ and ^232^K were found in all amino acid sequences from the 19RBR58-like cluster. Other Thai PCV2 sequences (PCV2a, b, d, and h) were ^133^ANAL^136^ or ^133^ATAL^136^. To our knowledge, PCV2 strains with ^133^HDAM^136^ have not been reported previously.

**Figure 2:**
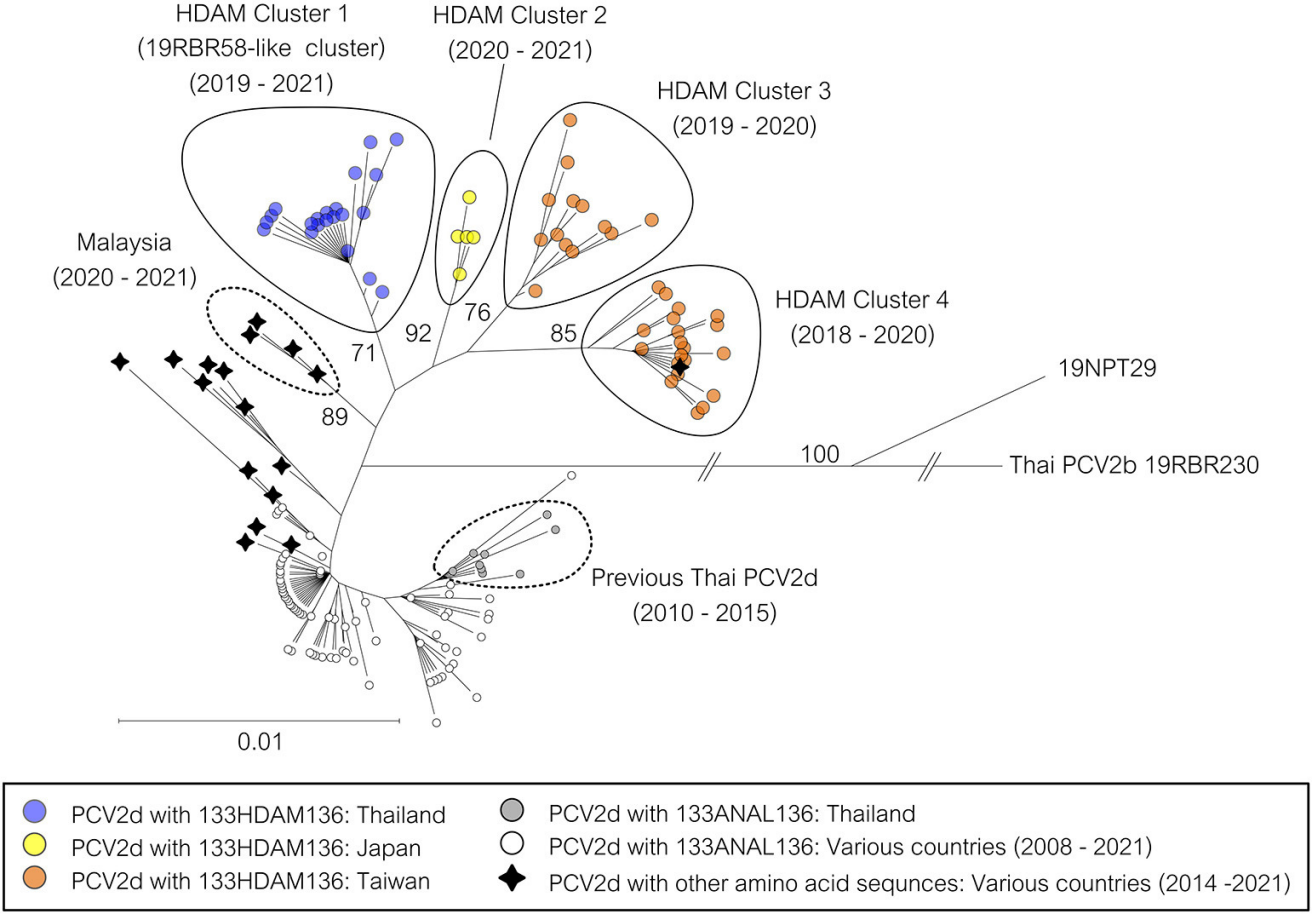
Phylogenetic tree of PCV2d ORF2 sequences from Thailand. The 158 complete ORF2 sequences were Thai PCV2d sequences in this study and the related PCV2d sequences from NCBI BLAST. A sequence of Thai PCV2b was used as an outgroup. The tree was constructed using the Neighbor-Joining method with the Maximum Composite Likelihood model and bootstrapping at 1,000 replicates. Node labels indicate bootstrap values. Specific branches were shortened, and the taxon position markers were adjusted to improve readability. A tree with each taxon label indicating the country of origin and the year of sample collection is available as Supplementary Figure 2.

NCBI BLAST function using 19RBR58 ORF2 (6^th^ February 2023) (https://blast.ncbi.nlm.nih.gov) was used to retrieve sequences with high similarity from GenBank database for further analysis (Supplementary Table 3). From the dataset, PCV2d strains with ^133^HDAM^136^ from Japan and Taiwan during the period of 2018–2020 were found. However, those sequences were direct submissions. Moreover, PCV2d with amino acid sequences other than HDAM, ANAL, and ATAL at the position 133–136 were also identified (Supplementary Table 3) such as HAAM and HNAM. Clustering of the 19RBR58-like viruses was shown in Figure 2.

### 3.3. The novel PCV2d variants was a parental strain of a PCV2b/2d recombinant virus

**Figure 3:**
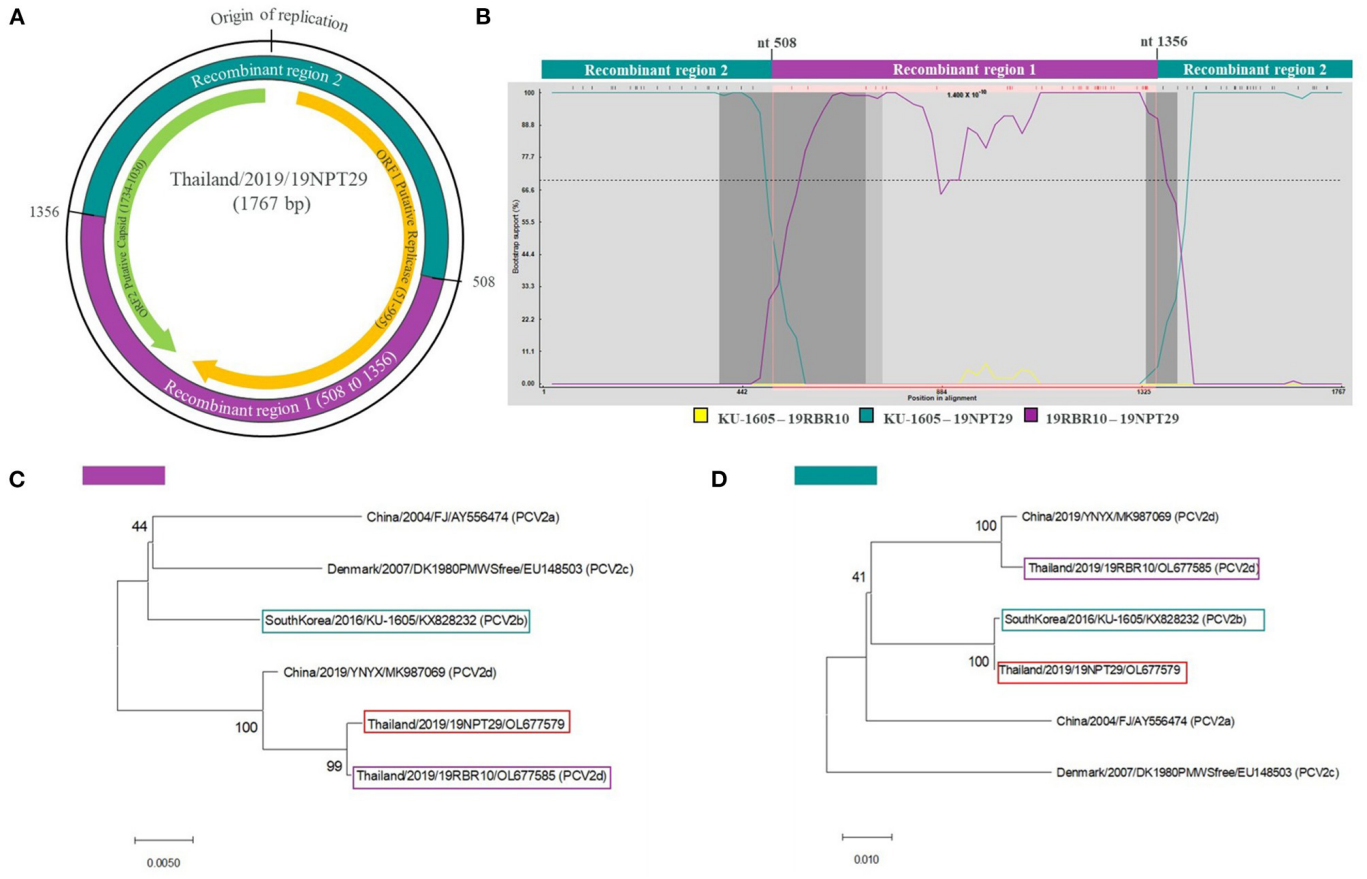
Recombination analysis of 19NPT29. Recombination analysis was done using RDP software. Recombination breakpoints were identified in ORF1 and ORF2 regions of the 19NPT29 genome, resulting in recombinant region 1 (purple shaded) and 2 (dark green shaded) **(A)**. Bootscanning analysis shows KU-1605 (South Korea) and 19RBR10 (Thailand, in this study) as parental strains **(B)**. The phylogenetic trees were constructed based on recombinant region 1 **(C)** and 2 **(D)** to confirm recombination event.

Recombination analysis using seven different methods provided strong statistical support (average *p* = 3.84 × 10^−9^) confirming that 19NPT29 is an intergenotypic recombinant virus of PCV2b and PCV2d. The analysis indicated that PCV2b strains (such as South Korea/2016/KU-1605-like viruses) and PCV2d strains (such as Thailand/2019/19RBR10-like viruses) were the potential parental strains involved in the recombination event. Notably, the parental PCV2d strain was also found within the 19RBR58-like cluster, and the presence of ^133^HDAM^136^ and ^232^K in the capsid protein was observed in 19NPT29. The putative recombination breakpoints were identified at nucleotide positions 508 (ORF1) and 1356 (ORF2) (Figure 3). The detection rate of the recombinant strain at the animal and farm levels was found to be 1.96% (1/51) and 2.08% (1/48), respectively.

## 4. Discussion

PCV2 is a major swine virus causing economic losses. Although vaccines have been widely used, vaccine failures and immune escaping mutation of PCV2 has been proposed (8). In this study, novel variants of PCV2d were identified providing a clue on the PCV2 evolution and epidemiology.

The prevalence of PCV2 in Thailand remained consistently high during the period of 2019–2020 compared to the period of 2009–2015. The prevalence at the animal level from 2009 to 2015 was 44.09% (18). In the current study, the prevalence increased to 54.2%. At the farm level, the prevalence from 2009 to 2015 was 80% (18), and in 2019–2020, it reached 81.4%. These findings suggests that PCV2 was still circulating, despite the implementation of PCV2 vaccines in Thailand. Unfortunately, the PCV2 vaccination status of each farm was not available in this study. Therefore, no conclusion can be made regarding the effect of the PCV2 vaccination and the overall PCV2 prevalence in Thailand.

In recent years, a genotype shift toward PCV2d can be observed in various countries, particularly in Asia. These countries include China (19), South Korea (20), Vietnam (21), Malaysia (22), and Thailand (18). In Thailand, the prevalence of PCV2d has been increasing since 2010, with only PCV2d detected by 2015 (18). However, in this study, a novel strain of PCV2d, which accounted for 69.77% of all the current Thai PCV2d, was identified. Therefore, this novel strain of PCV2d plays a crucial role in the prevailing PCV2d strain in Thailand during 2019–2020. Moreover, this finding suggests that it may serve as the starting point for the next genetic shift within PCV2d.

This study identified the dominance of novel PCV2d strains, the 19RBR58-like cluster, over the previously circulating PCV2 strains in Thailand. At position 133–136 of the capsid protein, the 19RBR58-like cluster was ^133^HDAM^136^ while other Thai PCV2 strains were ^133^ANAL^136^ or ^133^ATAL^136^. Notably, this region of amino acids resides in one of the antibody recognition domains (domain B) previously described (23, 24), i.e., domain A (aa 51–84), B (aa 113–139), C (aa 161–207), and D (aa 228–233). A single mutation at position 134, 135 or 136 has been shown to strongly reduce the neutralization activity (9). Therefore, the capsid protein with ^133^HDAM^136^ might render the 19RBR58-like cluster less susceptible to the antibodies from the previously circulating strains and the vaccines. In fact, PCV2 vaccination is widely implemented in Thailand (personal communication). The observed immune escaping mechanism is further supported by the rapid increase of the 19RBR58-like cluster. Prior to 2015, the 19RBR58-like cluster was not detected in Thailand, and there is a lack of sequence data from 2016 to 2018. Thus, it is possible that the emergence of the 19RBR58-like cluster occurred during the period of 2016–2018.

In addition to Thailand, this study also identified PCV2d sequences with ^133^HDAM^136^ from Japan and Taiwan (direct submission in GenBank). Interestingly, the strains carrying ^133^HDAM^136^ from each region formed a distinct cluster on the phylogenetic tree. This suggests that the current situation of these viruses may not be attributed to recent spreading between regions. Phylogenetic analysis further revealed that all the clusters harboring ^133^HDAM^136^ (Thailand, Japan, and Taiwan) likely share a common ancestor with PCV2d strains from Malaysia. At present, the prevalence of the ^133^HDAM^136^ PCV2d variants in Japan and Taiwan is unknown. Further investigations are needed to determine whether the prevalence of these PCV2d variants is high, similar to that observed in Thailand.

Recombinant viruses derived from PCV2d strains have been reported in various countries, including China, India, and South Korea (25–27). In this study, a recombinant PCV2d/PCV2b strain, named 19NPT29, was identified. Interestingly, ^133^HDAM^136^ was also found in the capsid protein of 19NPT29. It would be valuable to conduct further studies to investigate whether the presence of ^133^HDAM^136^ provides any advantages to the recombinant PCV2 strain, particularly in the case of inter-genotypic recombinants. Unfortunately, conducting further epidemiological studies on the 19NPT29-like viruses from the source farm is not possible as the farm is no longer operational.

The main limitation of this study was the absence of information regarding the vaccination status of the farms, along with the passive surveillance nature of the study. The observed mutation in the antibody recognition domain of the capsid protein within the 19RBR58-like cluster is suspected to have played a role in its emergence. Therefore, the information regarding the vaccination status would have been invaluable for interpreting the data and generating hypotheses for further studies on cross-protection. Furthermore, it is important to note that the samples used in this study were obtained from two diagnostic laboratories (CU-VDL and DLSTC), which may have led to potential underrepresentation of certain geographical regions. However, it is worth mentioning that this study managed to collect samples from all the high-pig-density regions in the country. Conducting active surveillance in the future may provide a more precise assessment of the prevalence of PCV2 and the PCV2d status.

In conclusion, this study reveals the presence of a novel PCV2d strain with ^133^HDAM^136^ in the capsid protein as the predominant PCV2 strain in Thailand. Additionally, a recombinant virus between PCV2b and the novel PCV2d was identified. The emergence of these novel PCV2d strains might have been influenced by both vaccination and the previously circulating viruses. Conducting active surveillance can provide a comprehensive understanding of PCV2 evolution and facilitate the implementation of early interventions against the emergence of novel strains.

## Data availability statement

The datasets presented in this study can be found in online repositories. The names of the repository/repositories and accession number(s) can be found in the article/ Supplementary material.

## Author contributions

CS, TN, and TJ collected the samples and did the PCR and sequencing. CS, RK, and RT analyzed the data and interpreted the results and prepared the manuscript. All authors helped in designing the research and contributed to the article and approved the submitted version.

## References

[1] Guo LJ Lu YH Wei YW Huang LP Liu CM. Porcine circovirus type 2 (Pcv2): genetic variation and newly emerging genotypes in China. Virol J. (2010) 7:273. 10.1186/1743-422X-7-273 20955622 PMC2967541

[2] Mankertz J Buhk HJ Blaess G Mankertz A. Transcription analysis of porcine circovirus (Pcv). Virus Genes. (1998) 16:267–76. 10.1023/A:1008022521329 9654680

[3] Nawagitgul P Morozov I Bolin SR Harms PA Sorden SD Paul PS. Open reading frame 2 of porcine circovirus type 2 encodes a major capsid protein. J Gen Virol. (2000) 81:2281–7. 10.1099/0022-1317-81-9-2281 10950986

[4] Olvera A Cortey M Segales J. Molecular evolution of porcine circovirus type 2 genomes: phylogeny and clonality. Virology. (2007) 357:175–85. 10.1016/j.virol.2006.07.047 16963096

[5] Grau-Roma L Fraile L Segales J. Recent advances in the epidemiology, diagnosis and control of diseases caused by porcine circovirus type 2. Vet J. (2011) 187:23–32. 10.1016/j.tvjl.2010.01.018 20211570

[6] Franzo G Segales J. Porcine circovirus 2 (Pcv-2) genotype update and proposal of a new genotyping methodology. PLoS ONE. (2018) 13:e0208585. 10.1371/journal.pone.0208585 30521609 PMC6283538

[7] Afolabi KO Iweriebor BC Okoh AI Obi LC. Global status of porcine circovirus type 2 and its associated diseases in Sub-Saharan Africa. Adv Virol. (2017) 2017:6807964. 10.1155/2017/6807964 28386278 PMC5366187

[8] Xiao CT Halbur PG Opriessnig T. Global molecular genetic analysis of porcine circovirus type 2 (Pcv2) sequences confirms the presence of four main Pcv2 genotypes and reveals a rapid increase of Pcv2d. J Gen Virol. (2015) 96:1830–41. 10.1099/vir.0.000100 25711965

[9] Huang L Sun Z Xia D Wei Y Sun E Liu C. Neutralization mechanism of a monoclonal antibody targeting a porcine circovirus type 2 cap protein conformational epitope. J Virol. (2020) 94:e01836–19. 10.1128/JVI.01836-19 32075932 PMC7163150

[10] Wang Y Feng Y Zheng W Noll L Porter E Potter M. A multiplex real-time pcr assay for the detection and differentiation of the newly emerged porcine circovirus type 3 and continuously evolving type 2 strains in the United States. J Virol Methods. (2019) 269:7–12. 10.1016/j.jviromet.2019.03.011 30904590 PMC7113677

[11] Fenaux M Halbur PG Gill M Toth TE Meng XJ. Genetic characterization of type 2 porcine circovirus (Pcv-2) from pigs with postweaning multisystemic wasting syndrome in different geographic regions of North America and development of a differential Pcr-restriction fragment length polymorphism assay to detect and differentiate between infections with Pcv-1 and Pcv-2. J Clin Microbiol. (2000) 38:2494–503. 10.1128/JCM.38.7.2494-2503.2000 10878032 PMC86951

[12] An DJ Roh IS Song DS Park CK Park BK. Phylogenetic characterization of porcine circovirus type 2 in Pmws and Pdns Korean pigs between 1999 and 2006. Virus Res. (2007) 129:115–22. 10.1016/j.virusres.2007.06.024 17706315

[13] Thompson JD Higgins DG Gibson TJ. Clustal W: improving the sensitivity of progressive multiple sequence alignment through sequence weighting, position-specific gap penalties and weight matrix choice. Nucleic Acids Res. (1994) 22:4673–80. 10.1093/nar/22.22.4673 7984417 PMC308517

[14] Hall TA. Bioedit: a user-friendly biological sequence alignment editor and analysis program for windows 95/98/Nt. Nucl Acids Symp Ser. (1999) 41:95–8.

[15] Tamura K Stecher G Peterson D Filipski A Kumar S. Mega6: molecular evolutionary genetics analysis version 60. Mol Biol Evol. (2013) 30:2725–9. 10.1093/molbev/mst197 24132122 PMC3840312

[16] Martin DP Lemey P Lott M Moulton V Posada D Lefeuvre P. RDP3: a flexible and fast computer program for analyzing recombination. Bioinformatics. (2010) 26:2462–3. 10.1093/bioinformatics/btq467 20798170 PMC2944210

[17] Lole KS Bollinger RC Paranjape RS Gadkari D Kulkarni SS Novak NG. Full-length human immunodeficiency virus type 1 genomes from subtype C-infected seroconverters in India, with evidence of intersubtype recombination. J Virol. (1999) 73:152–60. 10.1128/JVI.73.1.152-160.1999 9847317 PMC103818

[18] Thangthamniyom N Sangthong P Poolperm P Thanantong N Boonsoongnern A Hansoongnern P. Genetic diversity of porcine circovirus type 2 (Pcv2) in Thailand during 2009–2015. Vet Microbiol. (2017) 208:239–46. 10.1016/j.vetmic.2017.08.006 28888644

[19] Nan W Wu J Hu H Peng G Tan S Deng Z. Prevalence and genetic diversity of porcine circovirus type 2 in Northern Guangdong Province during 2016–2021. Front Vet Sci. (2022) 9:932612. 10.3389/fvets.2022.932612 36032297 PMC9399655

[20] Kwon T Lee DU Yoo SJ Je SH Shin JY Lyoo YS. Genotypic diversity of porcine circovirus type 2 (Pcv2) and genotype shift to Pcv2d in Korean pig population. Virus Res. (2017) 228:24–9. 10.1016/j.virusres.2016.11.015 27867029

[21] Doan HTT Do RT Thao PTP Le XTK Nguyen KT Hien NTT. Molecular genotypic analysis of porcine circovirus type 2 reveals the predominance of Pcv2d in Vietnam (2018-2020) and the association between Pcv2h, the recombinant forms, and vietnamese vaccines. Arch Virol. (2022) 167:2011–26. 10.1007/s00705-022-05517-4 35794492

[22] Tan CY Thanawongnuwech R Arshad SS Hassan L Fong MWC Ooi PT. Genotype shift of malaysian porcine circovirus 2 (Pcv2) from Pcv2b to Pcv2d within a Decade. Animals. (2022) 12:1849. 10.3390/ani12141849 35883396 PMC9311952

[23] Mahe D Blanchard P Truong C Arnauld C Le Cann P Cariolet R. Differential recognition of Orf2 protein from type 1 and type 2 porcine circoviruses and identification of immunorelevant epitopes. J Gen Virol. (2000) 81:1815–24. 10.1099/0022-1317-81-7-1815 10859388

[24] Lekcharoensuk P Morozov I Paul PS Thangthumniyom N Wajjawalku W Meng XJ. Epitope mapping of the major capsid protein of type 2 porcine circovirus (Pcv2) by using chimeric Pcv1 and Pcv2. J Virol. (2004) 78:8135–45. 10.1128/JVI.78.15.8135-8145.2004 15254185 PMC446101

[25] Wei C Lin Z Dai A Chen H Ma Y Li N. Emergence of a novel recombinant porcine circovirus type 2 in China: Pcv2c and Pcv2d recombinant. Transbound Emerg Dis. (2019) 66:2496–506. 10.1111/tbed.13307 31342637

[26] Jang G Yoo H Kim Y Yang K Lee C. Genetic and phylogenetic analysis of porcine circovirus type 2 on Jeju Island, South Korea, 2019–2020: evidence of a novel intergenotypic recombinant. Arch Virol. (2021) 166:1093–102. 10.1007/s00705-020-04948-1 33570666

[27] Parthiban S Ramesh A Karuppannan AK Dhinakar RG Johnson RJ Hemalatha S. Emergence of novel porcine circovirus 2 genotypes in Southern India. Transbound Emerg Dis. (2022) 69:1804–12. 10.1111/tbed.14158 34008351

