# Supplementary figures and images for: Emergence of novel porcine circovirus 2d strains in Thailand, 2019–2020
Source: Front Vet Sci. 2023 Jun 20;10:1170499. doi: 10.3389/fvets.2023.1170499 (PMC10318142; doi:10.3389/fvets.2023.1170499)

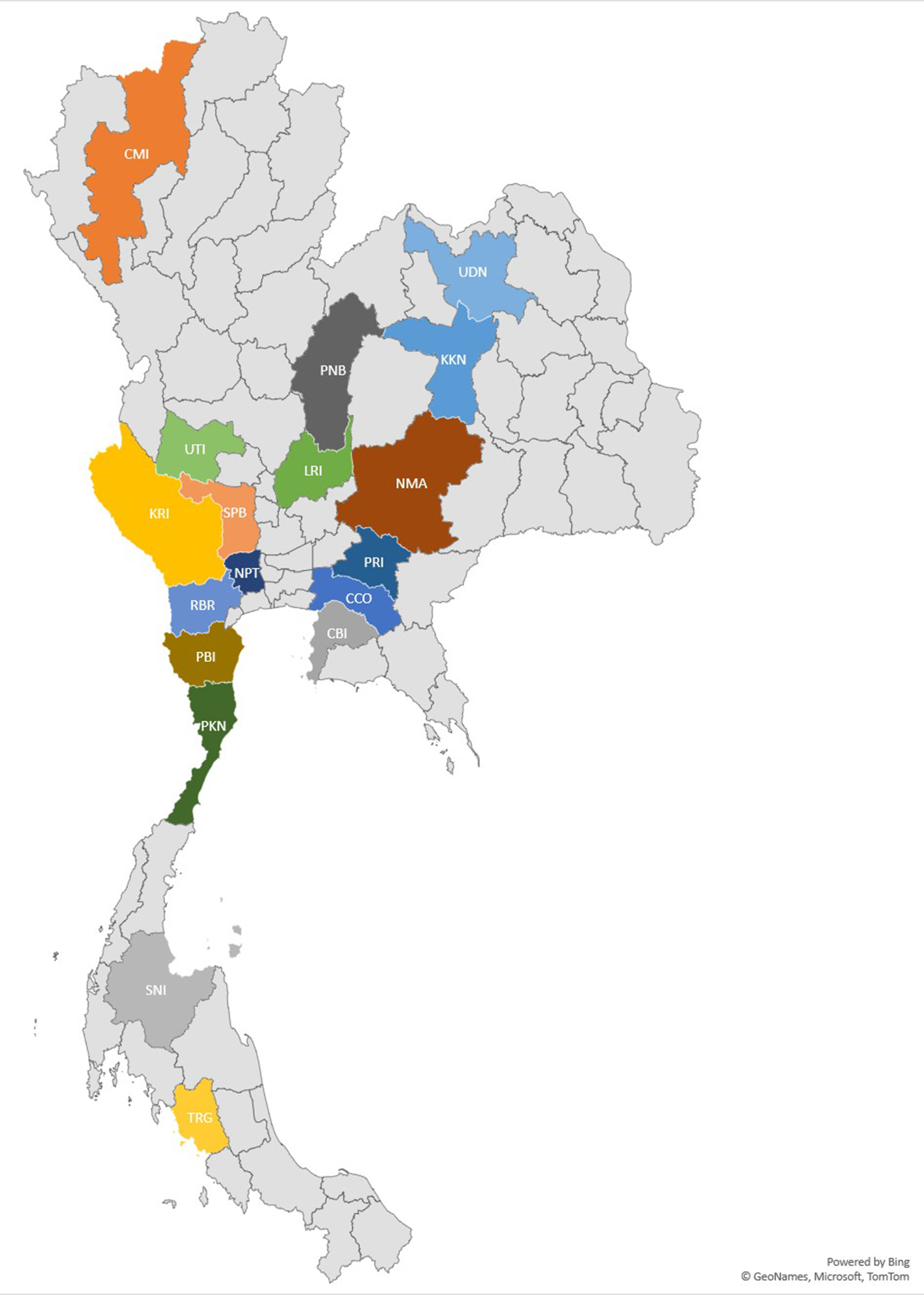

Supplement: Supplementary file 5 [file Image_1.JPEG]

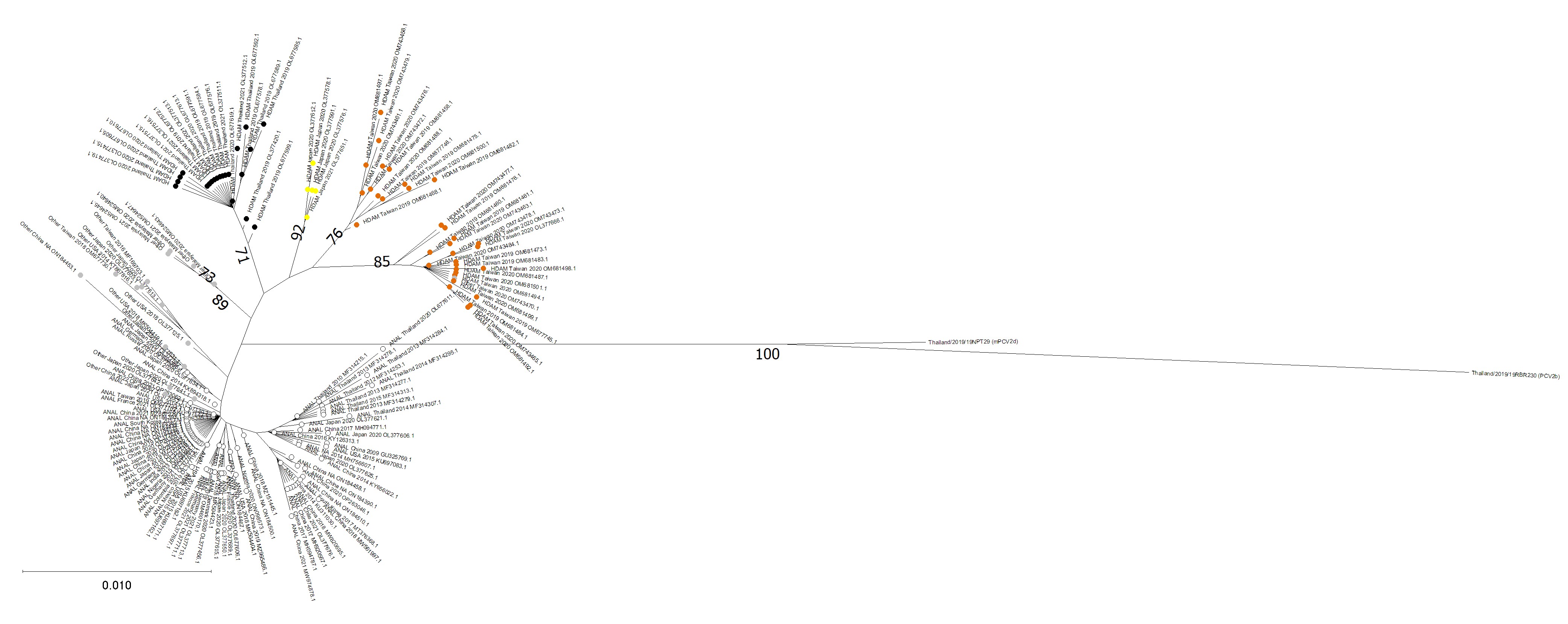

Supplement: Supplementary file 6 [file Image_2.JPEG]
